# Supplementary material for: Social Determinants of Health and College Food Insecurity
Source: Nutrients. 2024 May 4;16(9):1391. doi: 10.3390/nu16091391 (PMC11085391; doi:10.3390/nu16091391)
Supplement: Supplementary file 1 [file nutrients-16-01391-s001.zip › nutrients-2946510-supplementary.pdf]

Social Determinants of Health and College Food Insecurity

Supplementary Materials

Table S1. Mediation analysis of the effects of childhood food insecurity on health and well-being outcomes through college food insecurity using KHB method.

	Food stress	Difficulty concentrating on studies	More food challenges after COVID	General stress	Self-rated health	Perceived academic progress
Reduced (total effect)	1.076**	1.957**	0.722	0.733*	-0.924**	-0.333
	[0.443, 1.709]	[0.905, 3.009]	[-0.053, 1.497]	[0.115, 1.352]	[-1.531, -0.317]	[-0.997, 0.332]
Full (direct effect)	0.491	1.357**	0.493	0.481	-0.711*	-0.147
	[-0.146, 1.129]	[0.327, 2.386]	[-0.292, 1.278]	[-0.146, 1.108]	[-1.332, -0.091]	[-0.816, 0.522]
Diff (indirect effect)	0.585**	0.600**	0.229*	0.252**	-0.213*	-0.186*
	[0.246, 0.923]	[0.180, 1.020]	[0.033, 0.424]	[0.067, 0.438]	[-0.378, 0.047]	[-0.343, -0.029]
Mediation percent	54.34%	30.66%	31.67%	34.44%	23.03%	55.90%

Note: Unstandardized regression coefficients are shown; 95% Confidence Intervals in brackets. All models controlled for gender, sexual orientation, race/ethnicity, off-campus living, instate, first-generation, and study area. ** p<0.01, * p<0.05 (two-tailed test)

Table S2. P-values adjusted for multiple hypothesis testing correction compared to P-values from original models

	Food stress		Difficulty concentrating on studies		More food challenges after COVID		General stress		Self-rated health		Perceived academic progress	
	Model 1	Model 2	Model 1	Model 2	Model 1	Model 2	Model 1	Model 2	Model 1	Model 2	Model 1	Model 2
Childhood food insecurity												
Model p-value	0.0028	0.1306	0.0009	0.0098	0.0753	0.2185	0.0240	0.1330	0.0019	0.0246	0.4178	0.6673
Romano-Wolf p-value	0.0197	0.4197	0.0100	0.0553	0.2027	0.4197	0.0883	0.4197	0.0160	0.1077	0.4853	0.6927
College food insecurity												
Model p-value		0.0000		0.0000		0.0034		0.0001		0.0005		0.0028
Romano-Wolf p-value		0.0003		0.0013		0.0077		0.0017		0.0023		0.0077
Female												
Model p-value	0.8859	0.2090	0.7461	0.8695	0.5968	0.4621	0.0423	0.0201	0.0231	0.0096	0.4357	0.5965
Romano-Wolf p-value	0.9434	0.6285	0.9434	0.8880	0.9384	0.8624	0.2166	0.1123	0.1603	0.0776	0.8897	0.8624
LGBTQIA++												
Model p-value	0.0280	0.0140	0.0901	0.0463	0.0417	0.0388	0.0036	0.0025	0.0001	0.0000	0.4437	0.4765
Romano-Wolf p-value	0.1556	0.0863	0.2616	0.1649	0.1743	0.1649	0.0230	0.0173	0.0013	0.0003	0.4952	0.5002
Non-Hispanic black												
Model p-value	0.4959	0.9649	0.6069	0.2502	0.4514	0.6509	0.5104	0.7680	0.6863	0.8888	0.0445	0.0781
Romano-Wolf p-value	0.8880	0.9840	0.8880	0.6393	0.8880	0.9737	0.8880	0.9827	0.8880	0.9840	0.1423	0.2863
Hispanic												
Model p-value	0.4429	0.8351	0.5184	0.4777	0.2317	0.2824	0.1497	0.2012	0.6551	0.7791	0.0821	0.1239
Romano-Wolf p-value	0.8317	0.9517	0.8317	0.8437	0.6684	0.7334	0.5951	0.6831	0.8317	0.9517	0.4349	0.5795
Living off-campus												
Model p-value	0.0010	0.0123	0.0990	0.2983	0.4538	0.6283	0.3881	0.7684	0.4085	0.6477	0.3428	0.5805
Romano-Wolf p-value	0.0060	0.0780	0.4412	0.8537	0.8277	0.9690	0.8277	0.9690	0.8277	0.9690	0.8277	0.9690
Humanities/behavioral/social/health												
Model p-value	0.2319	0.8441	0.8163	0.7270	0.9368	0.6632	0.6960	0.3448	0.8423	0.5984	0.0592	0.0195
Romano-Wolf p-value	0.7348	0.9793	0.9957	0.9793	0.9957	0.9793	0.9900	0.8950	0.9957	0.9793	0.3336	0.1373
Business												
Model p-value	0.5430	0.8194	0.6913	0.8326	0.4793	0.4853	0.7111	0.6128	0.2349	0.2239	0.0705	0.0726
Romano-Wolf p-value	0.9357	0.9680	0.9357	0.9680	0.9357	0.9284	0.9357	0.9484	0.7473	0.7338	0.3583	0.3972

Notes: Model p-values from original regressions and Romano-Wolf stepdown adjusted p-values are shown. The regression models estimated for this table are the same as those for Table 3 and Table 4. Only variables that have a significant effect on at least one outcome are shown in the table.
